# Dendritic Cells and Their Multiple Roles during Malaria Infection

**DOI:** 10.1155/2016/2926436

**Published:** 2016-03-24

**Authors:** Kelly N. S. Amorim, Daniele C. G. Chagas, Fernando B. Sulczewski, Silvia B. Boscardin

**Affiliations:** ^1^Laboratory of Antigen Targeting to Dendritic Cells, Department of Parasitology, Institute of Biomedical Sciences, University of São Paulo, 05508-000 São Paulo, SP, Brazil; ^2^National Institute for Science and Technology in Vaccines, 31270-910 Belo Horizonte, MG, Brazil

## Abstract

Dendritic cells (DCs) play a central role in the initiation of adaptive immune responses, efficiently presenting antigens to T cells. This ability relies on the presence of numerous surface and intracellular receptors capable of sensing microbial components as well as inflammation and on a very efficient machinery for antigen presentation. In this way, DCs sense the presence of a myriad of pathogens, including* Plasmodium* spp., the causative agent of malaria. Despite many efforts to control this infection, malaria is still responsible for high rates of morbidity and mortality. Different groups have shown that DCs act during* Plasmodium* infection, and data suggest that the phenotypically distinct DCs subsets are key factors in the regulation of immunity during infection. In this review, we will discuss the importance of DCs for the induction of immunity against the different stages of* Plasmodium*, the outcomes of DCs activation, and also what is currently known about* Plasmodium* components that trigger such activation.

## 1. Introduction

Malaria is the most important protozoan parasitosis in humans. It afflicts millions of people annually causing an expressive burden, mainly in tropical countries.* Plasmodium* has a very complex life cycle, and its different stages alternate between the vertebrate host and the mosquito. The vertebrate infective stages are able to move around in the skin but also traverse and infect cells in tissues. During this journey,* Plasmodium* interacts with DCs that are responsible for the initiation of adaptive immune responses. The interactions among DCs and the parasite are complex and may shape the outcome of the anti-*Plasmodium* immune responses.

## 2. Dendritic Cells and Their Role in the Induction of Immune Responses

DCs are a distinct lineage of mononuclear phagocytic cells specialized in antigen presentation. They show excellent ability to capture, process, and present antigens to T cells [1], directly activate B cells [2], and are also involved in the amplification of innate immune responses, such as activation of NK cells [3, 4]. Once in contact with a pathogen, DCs undergo a process known as maturation that culminates with efficient antigen presentation and cytokine production. Cytokines produced by DCs become part of the microenvironment that induces immune responses capable of stimulating the development of effector T lymphocytes [5]. In addition, DCs are involved in tolerance development in the thymus by negative selection of autoreactive lymphocytes [6] and in the periphery, where they present self-antigens in the absence of inflammation [7]. This entire range of DC functions is associated with their ability to recognize pathogen- or damage-associated molecular patterns (PAMPs or DAMPs, resp.) through pattern recognition receptors (PRRs) [8].

Different classes of PRRs were discovered in the last decades and include membrane anchored receptors such as toll-like receptors (TLRs) [9] and C-type lectin receptors (CLRs) [10], besides the cytoplasmic nucleotide-binding oligomerization domain- (Nod-) like receptors (NLRs), RIG-I-like receptors (RLRs), and AIM-2-like receptors [11, 12], as well as a family of enzymes that function as intracellular sensors of nucleic acids, including OAS proteins and cGAS [12]. These PRRs are capable of triggering complex intracellular signals that stimulate DC maturation, increase the expression of major histocompatibility complex (MHC) and costimulatory molecules, and promote proinflammatory cytokines expression [13, 14]. Thus, in a context of infection and inflammation, DCs can identify the presence of pathogens through PRRs and induce adaptive immune responses [13].

**Figure 1:**
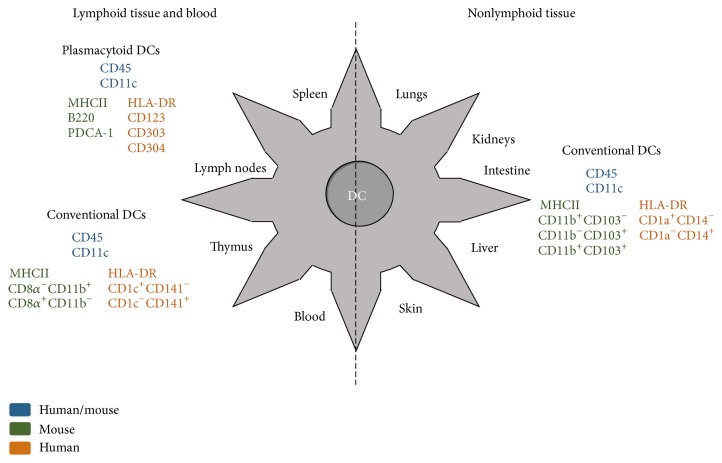
Most common DC surface markers used to differentiate conventional from plasmacytoid DCs and lymphoid tissue/blood from nonlymphoid tissue resident DCs. The colors indicate if a specific marker is expressed only in humans (orange), only in mice (green), or in both (blue) either in humans or in mice. Langerhans cells (LCs) are not represented. Based on [15, 125, 126].

DCs can be subdivided into different subsets based on the expression of different surface molecules (Figure 1). Human and mouse DCs normally express CD45, CD11c, and MHC class II (MHCII). In mice, the CD11c and MHCII molecules are expressed in all DC subsets with different intensities, and other markers such as CD11b, CD8*α*, CD103, and B220 can be used to distinguish the subsets even further. DCs expressing B220 and intermediate amounts of CD11c are known as plasmacytoid dendritic cells (pDCs). DCs expressing high levels of CD11c are known as conventional dendritic cells (cDCs) or myeloid dendritic cells (mDCs) and can be subdivided into CD8*α* ^+^CD11b^−^ and CD8*α* ^−^CD11b^+^ in lymphoid tissues and CD103^+^CD11b^−^, CD103^+^CD11b^+^, and CD103^−^CD11b^+^ in nonlymphoid tissues [15]. Langerhans cells (LCs) are DCs that populate the epidermal layer of skin and, different from cDCs, undergo a unique differentiation process [16].

The CD8*α* ^+^CD11b^−^ cDCs are efficient in antigen capture and presentation to CD4^+^ T cells in the context of MHCII molecules but have been mainly associated with antigen presentation to CD8^+^ T cells [17]. They are also able to cross-present exogenous antigens in MHC class I (MHCI) molecules, promoting activation of CD8^+^ T cells [18]. On the other hand, CD8*α* ^−^CD11b^+^ DCs are extremely efficient in presenting antigens to CD4^+^ T cells, polarizing them to Th2 or Th17 that in turn help B cells to produce antibodies [19].

As mentioned above, cDCs can reside in lymphoid and nonlymphoid organs. Normally, cDCs residing in nonlymphoid organs such as skin, lungs, or gut are more frequently in contact with antigens derived from pathogens. They capture antigens in the periphery and then move to lymphoid organs, where they present them to T cells. During migration, DCs mature and are able to prime lymphocytes, initiating the adaptive immune response [20]. Evidence has shown that the CD103^+^ DCs are the nonlymphoid organ counterparts of the CD8*α* ^+^ DCs, as they have a similar expression profile and can also efficiently perform cross-presentation and CD8^+^ T cell activation [15, 21].

On the other hand, pDCs express lower levels of MHCII and are much less efficient in inducing proliferation of T lymphocytes [22, 23]. However, during inflammation pDCs can be activated and differentiated into a specific DC subtype in spleen with higher capacity to induce T cell activation [24–26].

There are relatively fewer studies on human DCs when compared to mice, mainly because of the difficulty in isolating them from human tissues. In this way, most of our knowledge involves skin and blood DCs [15]. As in mice, human DCs can be divided into mDCs (or cDCs) and pDCs. In the blood and in lymphoid tissues, mDCs are CD3^−^CD14^−^CD19^−^CD20^−^CD56^−^HLA-DR^+^CD11c^+^ [27] and can be further subdivided into two subsets that express CD1c (BDCA1) or CD141 (BDCA3) [28]. On the other hand, pDCs are CD3^−^CD14^−^CD19^−^CD20^−^CD56^−^HLA-DR^+^CD11c^+^CD303(BDCA2)^+^CD304(BDCA4)^+^ [29]. In the skin, three populations can be found: CD1a^+^CD14^−^ DCs, CD1a^−^CD14^+^ DCs, and LCs [15, 30]. Their functions in the steady state or during inflammatory/infectious diseases are beginning to be elucidated only recently [31].

## 3. Malaria

Malaria is an infection caused by protozoa that belong to the phylum Apicomplexa, genus* Plasmodium*. This unicellular parasite is transmitted to humans through the bite of an infected mosquito. Approximately 200 million cases of this disease are reported annually causing half a million deaths. Most of the deaths occur among children living in Africa. Malaria is prevalent in tropical and subtropical regions and is currently endemic in parts of America and parts of Asia and in Sub-Saharan Africa. In 2013, 97 countries reported endemic malaria [32].

There are five species of* Plasmodium* that infect humans:* P. falciparum*,* P. vivax*,* P. malariae*,* P. knowlesi*, and* P. ovale* [32]. The parasitic cycle begins when infected mosquitoes transfer sporozoites (the infective forms of* Plasmodium*) during the blood meal. These forms can remain in the skin for hours, slowly reaching circulation [33, 34]. In the circulatory system, sporozoites are carried to the liver, where they invade and replicate within hepatocytes [35]. Replication gives rise to merozoites that are released from the liver in structures named merosomes and can subsequently invade erythrocytes [36]. This phase of the cycle is known as erythrocytic phase and is when malaria symptoms start. At this stage, schizogony is repeated at specific intervals depending on the species and the febrile seizures correlate with the release of merozoites in the circulation. After a few days, some merozoites that infect erythrocytes give rise to male and female gametocytes that once taken up by the mosquito continue the cycle in the invertebrate host.

Severe cases of malaria are generally caused by* P. falciparum.* Infection with this parasite may progress to cerebral malaria, and infected individuals often present neurological symptoms such as convulsions and coma. In addition, patients with severe malaria may also present abnormal posture, respiratory syndrome, severe anemia, and multiple organ failure [37, 38]. The fact that severe malaria is usually associated with* P. falciparum* may be related to the potential of this species to produce hyperparasitemia. On the other hand,* P. falciparum* is the only species that clearly produces alterations in the microcirculation, allowing the parasite to escape destruction in the spleen. For example, erythrocytes infected with* P. falciparum* have the ability to adhere to the microvasculature. This phenomenon is known as cytoadherence and is mediated by molecules expressed by the infected erythrocyte that are able to bind to a series of endothelial receptors [39], such as CD36 and ICAM-1 [40, 41]. In addition, infected erythrocytes are able to bind to other infected and also noninfected erythrocytes, in a phenomenon known as rosetting. In this case, there is formation of cell aggregates that also interfere with the microcirculation [42].

The immunologic memory generated during infection with* Plasmodium* spp. is most often transient and restricted to patients living in endemic areas due to frequent exposure to the parasite by bites of infected mosquitoes [43]. In other words, naturally acquired immunity is not sterilizing and requires the persistence of the parasite to maintain the population of memory cells [44]. Several evidences suggest that naturally acquired protective immunity against malaria is obtained after successive infections [45]. Children intensely exposed to transmission have successive clinical episodes of malaria. With increasing age, clinical symptoms are less pronounced, although individuals may have high blood parasitemia [46]. In general, naturally acquired immunity is partly strain specific and primarily leads to a reduction of mortality rates and incidence of complications and later to a decrease in the incidence of disease. Finally, this naturally acquired immunity leads to a drop in parasitemia to low or even undetectable levels by conventional detection methods [47].

There is evidence that protective immune responses against malaria (sporozoites or blood stages) are initiated when antigen-presenting cells, DCs or macrophages, internalize the parasite and process and present its antigens to T cells via MHCI (through cross-presentation) or MHCII in a proinflammatory environment in which IL-12, TNF*α*, and IFN*γ* are produced. During the blood stage infection, it was shown that these cytokines act synergistically activating macrophages to produce reactive oxygen species (ROS) and nitrogen leading to parasite death [48, 49]. CD8^+^ T cells have also been implicated in the protective immune response against sporozoites of* Plasmodium* [50, 51]. Several studies have shown that the CD8^+^ T cells play a vital role in immunity against the preerythrocytic phase of* Plasmodium* both in mice [52] and in humans [53]. CD8^+^ T cells eliminate infected hepatocytes and are capable of producing TNF*α* and IFN*γ* [54]. The role of CD4^+^ T cells during* Plasmodium* infection has also been extensively studied in humans and mice. They seem to be particularly important during the erythrocytic phase, when IFN*γ* producing CD4^+^ T cells are required for the elimination of iRBCs. In addition, follicular helper T (Tfh) cells were also shown to be pivotal for the activation of antibody producing B cells [55].

Our understanding of the immunity and immunoregulation that develop during malaria is still incomplete. DCs act in the immune response to activate and/or regulate production of proinflammatory or regulatory cytokines that may have fundamental roles in regulating the acquisition of protection or in the exacerbated response observed in severe malaria patients. In the following sections, we will discuss what is known about DC participation during* Plasmodium* infection. Much of this knowledge was generated using mouse models that can only be infected with murine* Plasmodium* species. Despite that limitation, mouse models have helped us to understand how DCs influence the anti-*Plasmodium* response.

## 4. Role of Dendritic Cells during* Plasmodium* Infection

DCs ability to present antigens during malaria infection was recently reviewed [48]. As mentioned above, DCs are responsible for T cell priming and thus regulate the development of adaptive immune responses [56]. DC function has been extensively studied during infection with different species of* Plasmodium*, and, in some aspects, contradictory data was obtained. The reasons for such contradictions could be ascribed to the use of different* Plasmodium* species and stages, as well as differences in the DC activation status. We will discuss the data in more detail in the sections ahead.

### 4.1. DCs during Acute* Plasmodium* Infection in Humans

The first study analyzing DC function directly in the context of* P. falciparum* infection was reported by Urban et al. These authors incubated monocyte-derived DCs with infected red blood cells (iRBCs) and showed that they were able to bind to human DCs and inhibit their maturation, reducing their ability to stimulate T cell responses [57]. These results were questioned by Elliott et al. when they showed that in fact inhibition of DC maturation was only obtained when iRBCs were used in a 100 : 1 iRBC : DC ratio and was not contact dependent. In addition, when a lower dose of iRBC (10 : 1 ratio) was used, DCs matured efficiently and activated autologous T cell proliferation [58]. In a field study with Kenyan children, BDCA3^+^ DCs were significantly increased during acute infection, while CD1c^+^ DC numbers were unaltered when compared to healthy individuals [59, 60]. In addition, an association between increased numbers of circulating BDCA3^+^ DCs and severe human malaria was also observed [60, 61].

Although more prevalent, infection with* P. vivax* is more benign and less studied. However, a few studies were performed in an attempt to examine DC status and function during acute and symptomatic* P. vivax* infection. For example, the numbers of pDCs (HLA-DR^+^CD123^+^) and mDCs (HLA-DR^+^CD11c^+^) were evaluated in infected individuals from Thailand and Brazil, and a decrease in the mDCs/pDCs ratio was observed in both studies [62, 63]. In addition, 1/3 of the* P. vivax*-infected Brazilian patients showed low surface expression of CD86 [62]. A similar reduction in CD86 levels, as well as in CD83 and HLA-DR, was observed in Indonesian patients infected with* P. falciparum* and* P. vivax* [64]. This reduction correlated with an increase in DCs spontaneous apoptosis and impairment in their ability to capture, mature, and present antigens to T cells [64]. Interestingly, when patent asymptomatic patients were studied, HLA-DR expression was preserved in groups infected with either* P. vivax* or* P. falciparum* [65]. This result may indicate that DC function is preserved in patients that are infected but do no present symptoms, suggesting that functional DCs are important for the maintenance of clinical, but not parasitological, immunity.

### 4.2. Are DCs Capable of Inducing T and B Cell Responses during* Plasmodium* Infection?

**Figure 2:**
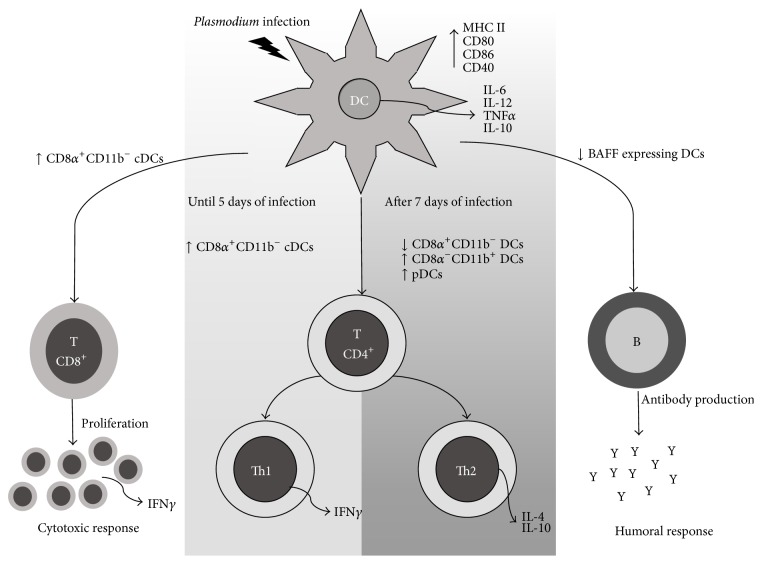
*Plasmodium* infection activates different DCs subsets. Infection with different stages of* Plasmodium* activates DCs that in turn start producing proinflammatory (IL-6, IL-12, and TNF*α*) but also regulatory (IL-10) cytokines. The CD8*α* ^+^CD11b^−^ DCs are responsible for priming CD8^+^ T cell responses against intrahepatic forms, while both cDCs subsets (CD8*α* ^+^CD11b^−^ and CD8*α* ^+^CD11b^+^) play a role in the activation of CD4^+^ T cell responses that can lead to an inflammatory or regulatory outcome, depending on the timing of infection. pDCs also play a role in the induction of a more regulatory CD4^+^ T cell response. The number of BAFF expressing DCs is reduced during* Plasmodium* infection, and that may reduce their ability to support B cell differentiation directly. Based on [69, 70, 78–80, 82, 85, 86, 91].

Human and murine species of* Plasmodium* have been used in studies designed to harness the participation of DCs in the induction of immunity against the preerythrocytic stages. DCs pulsed with sporozoite extracts were shown to elicit specific killing of* P. vivax* exoerythrocytic stages within infected hepatocytes [66], while DCs pulsed with a well-characterized CD8^+^ T cell epitope derived from the* P. yoelii* circumsporozoite protein reduced the liver burden in BALB/c mice after a sporozoite challenge [67]. This CD8^+^ T cell response was abrogated when DCs were depleted* in vivo* [68]. Moreover, after an infectious mosquito bite, the CD8*α* ^+^CD11b^−^ DCs located in the draining lymph nodes were shown to be the DC subset responsible for CD8^+^ T cell priming* in vivo* [69, 70] (Figure 2). In the* P. berghei* model, DCs pulsed with irradiated sporozoites were able to similarly prime central memory CD8^+^ T cells when compared to DCs primed with untreated sporozoites. However, irradiation enhanced sporozoites' ability to prime effector CD8^+^ T cells capable of producing IFN*γ*. In this particular study, the fine specificity of CD8^+^ T cells was not evaluated [71]. A more detailed study showed that the two major splenic DCs subsets (CD8*α* ^+^CD11b^−^ and CD8*α* ^−^CD11b^+^) induced IFN*γ* producing CD8^+^ T cells specific for the circumsporozoite protein, the major sporozoite surface protein [72]. In addition, the CD8^+^ T cell protective response against a genetically modified* P. yoelii* strain was shown to be dependent on effective DC maturation obtained through CD40 signaling [73]. The fact that DCs are able to induce CD8^+^ T cell responses against antigens expressed in the preerythrocytic stage seems undisputable. However, an observation made by Ocaña-Morgner et al. added another layer of complexity to the picture. These authors observed that DCs from mice previously infected with* P. yoelii* strain 17XNL and undergoing erythrocytic cycle presented an immature phenotype and were unable to initiate CD8^+^ T cell responses to subsequent liver-stage antigens [74]. An unidentified soluble factor released by the iRBC seemed to be responsible for the inhibition of DCs maturation [75]. Cross-presentation to CD8^+^ T cells was also inhibited during active* P. berghei* blood infection [76] and was mainly dependent on the CD8*α* ^+^CD11b^−^ DCs subset [77]. Taken together, these results suggest that blood stage infection with* Plasmodium* can impair the development of an effective CD8^+^ T cell response against the liver stages, which then could clear the parasite upon reinfection.

The role of DCs in the activation of CD4^+^ T cell responses was also evaluated during* P. yoelii* infection (Figure 2). DCs derived from infected mice (day 6) presented higher expression of the surface costimulatory molecules CD80 and CD40 and were able to efficiently present antigens to CD4^+^ T cells that in turn produced higher levels of IL-2, IFN*γ*, and TNF*α*. Production of these cytokines required DC-derived IL-12 [78, 79].

Infections with* P. chabaudi* have also shed light on the role of DCs during malaria.* In vitro* studies showed that* P. chabaudi* schizonts induced bone marrow-derived DCs to express MHCII and costimulatory molecules and to produce IL-6, IL-12, and TNF*α* [80]. An* in vivo* study showed that DCs migrated from the marginal zone of the spleen into the T cell area within 5 days after infection, and by day 7 an increase in costimulatory molecules was observed [81]. The role of CD8*α* ^+^CD11b^−^ and CD8*α* ^−^CD11b^+^ DCs subsets in antigen presentation and specific CD4^+^ T cell activation was also investigated. Despite the fact that both subsets induced IFN*γ* production, only the CD8*α* ^−^CD11b^+^ DCs isolated at the infection peak (day 7) were able to induce proliferation of* Plasmodium*-specific Tg CD4^+^ T cells and considerable amounts of IL-4 and IL-10 [82], indicating that this subset could be responsible for the switch in the balance from the proinflammatory Th1 response seen in the first few days to a more pronounced Th2 response. However, on days 10 and 13 after* P. chabaudi* infection, the CD8*α* ^−^CD11b^+^ DCs were no longer able to induce CD4^+^ T cell proliferation or cytokine production due to downregulation of costimulatory molecules and IL-12 expression [83]. Nevertheless, the* in vivo* CD4^+^ T cell response later recovers and the mice are able to control infection, and at this stage antibodies seem to play an important role in the parasitemia control [55, 84]. pDCs were also studied in this model and, despite an increase in their numbers during infection, they played no role in CD4^+^ T cell activation or in the control of infection [85]. On the other hand, in a* P. yoelii* model of infection, pDCs numbers increased by day 6 and remained high until at least day 14, and they were able to induce IL-10-expressing CD4^+^ T cells [86]. Taken together the results discussed above suggest that different DC subsets have different functions during the blood stage* Plasmodium* infection, with cDCs involved in the induction of the proinflammatory response and pDCs accounting for a more balanced response at a later stage (Figure 2).

As reported for* P. falciparum* [57] and* P. yoelii* [74], DCs were also able to bind and internalize* P. chabaudi* iRBC. This phenomenon was shown to be partially dependent on actin polymerization, and the iRBCs uptake was again associated with increased expression of MHCII and costimulatory molecules, IL-12 production, and stimulation of CD4^+^ T cell proliferation and IFN*γ* production [87]. Moreover, a recent study using intravital microscopy in mice showed that splenic DCs not only interact with CD4^+^ T cells in T cell rich areas and in the red pulp but also very actively phagocytose iRBCs, contributing directly to their elimination during acute infection [88].

The function of DCs in the development of cerebral malaria was also assessed using a model of* P. berghei* ANKA strain infection in C57BL/6 mice. In this setting, cDCs were shown to play a major role in the induction of cerebral malaria [89]. A more detailed study showed that the CD8*α* ^+^ and CD103^+^ DCs subsets are essential to induce the pathogenic CD8^+^ T cells responsible for lethal brain inflammation during* P. berghei* ANKA infection [60]. A recent set of experiments showed that the numbers of cDCs and pDCs were drastically reduced during* P. berghei* ANKA infection, through a mechanism that involved activation of caspase-3 and induction of DC apoptosis. In this particular case, the function of the remaining DCs was not evaluated [90].

DCs also play an important role in direct B cell activation (Figure 2). This happens because they produce a cytokine known as B cell activating factor (BAFF) that enhances B cell differentiation and survival [2]. During* P. yoelii* infection, there is a decrease in the percentage of DCs expressing BAFF, resulting in a reduction of their ability to support memory B cell differentiation into antibody secreting cells [91].

Finally, besides T and B cells, DCs are also able to interact with innate immune cells such as NK and *γδ* T cells during* Plasmodium* infection. The depletion of NK cells during* P. berghei* ANKA infection led to a significant reduction in DC-mediated CD8^+^ T cell priming but did not affect CD4^+^ T cells. It seems that NK cells stimulate DCs to produce IL-12 that in turn is required for optimal T cell priming. The effect of DCs on NK cell function was also evaluated, and DC depletion reduced NK cell-mediated IFN*γ* responses to this* Plasmodium* species [92]. In the same way, *γδ* T cells can also communicate with DCs and do so when they express CD40L and produce IFN*γ* that in turn enhance DC activation [49].

The data discussed above indicates that DCs may play distinct roles during* Plasmodium* infection, promoting either activation of protective immune responses or exacerbation of pathology.

### 4.3. How Do DCs Recognize* Plasmodium* Components?

The expression of TLRs on DCs was studied in patients infected with* P. falciparum*. Patients with mild and severe forms of the disease displayed increased surface expression of TLR2 and TLR4 on mDCs and decreased intracellular expression of TLR9 on pDCs, when compared to healthy controls [93]. Despite this decreased TLR9 expression on pDCs, another study showed that the TLR9-MyD88 signaling pathway was required for pDC activation upon stimulation using schizonts or soluble schizont extracts [94]. The possible ligand for this pathway will be discussed below. A study using* P. chabaudi* infected mice showed that DCs from MyD88 knockout mice, but not from TLR2, TLR4, TLR6, TLR9, or CD14 knockout mice, were unable to produce proinflammatory cytokines and induce CD4^+^ T cell responses [95]. This result indicates that the adaptor molecule MyD88 is required, but different ligands may be signalizing through different TLRs.

As mentioned before, DCs express different classes of PRRs capable of recognizing PAMPs derived from a vast array of pathogens [8]. In the case of* Plasmodium*, three PAMPs have been more extensively studied: hemozoin, immunostimulatory nucleic acid motifs, and glycosylphosphatidylinositol (GPI) anchors [96].

When* Plasmodium* invades the RBC, it degrades hemoglobin as a source of amino acids, which in turn releases heme that is potentially toxic. To survive, the parasite detoxifies heme into hemozoin using an enzyme named Heme Detoxification Protein (HDP) [97]. Contrasting results have been obtained and the role of hemozoin is still a matter of debate. Initially, hemozoin was shown to be present inside macrophages and circulating monocytes during* P. falciparum* infection, reducing their ability to phagocytose other particles or generate oxidative burst [98]. Human monocytes loaded with hemozoin and then* in vitro* differentiated into DCs presented impaired surface expression of MHCII and costimulatory molecules [99, 100], while monocyte-derived DCs incubated with synthetic hemozoin upregulated costimulatory molecules and released proinflammatory (IL-6) and anti-inflammatory (IL-10 and TNF*α*) cytokines, but not IL-12, leading to suboptimal T cell activation [101, 102]. In a* P. chabaudi* model, hemozoin-containing DCs were unable to fully activate T cells that in consequence did not cluster or migrate to lymphoid organ follicles [103, 104].

Different results showed that* P. falciparum* iRBC hemozoin, but not -hematin (a synthetic hemozoin), was able to induce human monocyte-derived DCs to upregulate costimulatory molecules (CD83, CD86, and CD1a) and produce IL-12 [105]. A follow-up study by the same group showed that this effect was dependent on TLR9 activation followed by MyD88 signaling but independent of TLR2, TLR4, TLR7, or TRIF [106]. In contrast with these authors, Parroche et al. showed that hemozoin is not a direct ligand for TLR9; instead it functions as a carrier for plasmodial DNA that is phagocytosed by DCs and carried to intracellular compartments [107]. Finally, Wu et al. argued that hemozoin was not a TLR9 ligand for DCs and did not function as a DNA carrier into cells. Instead, their results showed that a protein-DNA complex was the parasite's component responsible for the DC activation through TLR9 signaling. Protein-DNA complex formation was essential for the entry of parasite DNA into DCs leading to TLR9 recognition [108]. In addition, hemozoin was also found to activate the NLRP3 inflammasome during experimental malaria infections [109, 110].

*P. falciparum* genome is specially rich in AT-motifs. It was recently shown that these motifs have immunostimulatory properties and are able to induce type I IFNs. In fact,* Plasmodium* iRBCs triggered type I IFN production in macrophages [111]. DCs were not directly tested, but there is a very good possibility that they are activated in the same way.

GPI anchors are essential for* Plasmodium* survival and link different proteins to the parasite surface.* P. falciparum* merozoite-derived GPI anchors induced the production of proinflammatory cytokines and nitric oxide by macrophages [112, 113] mainly in a TLR2-dependent way but also, to a lesser extent, in a TLR4-dependent way [114], which requires CD36 cooperation [115].* P. falciparum* GPIs' ability to activate DCs was evaluated more recently, and a study showed that TLR2-signaling was also important for DCs activation and induction of TNF*α* and IL-12 production. As observed for macrophages, CD36 also collaborated with TLR2 [116].

The participation of different TLRs was also evaluated in a* P. berghei* ANKA model of cerebral malaria. Results showed that mice deficient in TLR1, TLR2, TLR3, TLR4, TLR6, TLR7, or TLR9 and their adapter proteins MyD88, TIRAP, and TRIF were as susceptive to cerebral malaria as their wild-type counterparts [117, 118]. In contrast to these results, another group showed the contrary: cerebral malaria pathogenesis seems to be mediated by MyD88 signaling and its absence increases survival [119]. DCIR—a CLR that recognizes carbohydrates—deficient mice are resistant to cerebral malaria and present less CD8^+^ T cell infiltration and inflammation in their brains when compared to wild-type mice [120].

In addition to PAMPs,* Plasmodium* infected erythrocytes were also shown to accumulate uric acid, a very potent endogenous danger-associated molecular pattern (DAMP). Uric acid precipitates accumulated within* P. falciparum* and* P. vivax* iRBC are released by cell rupture and induce the maturation of human dendritic cells* in vitro* [121]. Taken together, these results indicate that DCs sense and are able to respond to different* Plasmodium* PAMPs and also to DAMPs that are generated during infection.

## 5. Harnessing DCs for the Development of Vaccines against Malaria

DCs have also been studied as targets for the development of vaccines against malaria. The circumsporozoite protein (CSP)—the major sporozoite surface protein—from* P. yoelii* was fused to a monoclonal antibody capable of binding to a CLR—known as DEC205—expressed on the surface of the CD8*α* ^+^CD11b^−^ DCs subset. The administration of low doses of the *α*DEC205-CSP monoclonal antibody in the presence of a DCs maturation stimulus—*α*CD40+polyinosinic:polycytidylic acid, poly(I:C)—was able to induce IFN*γ* producing CD4^+^ and CD8^+^ T cells, besides specific anti-CSP antibodies [122]. The use of an anti-human DEC205 monoclonal antibody fused to the* P. falciparum* CSP together with poly(I:C) to immunize nonhuman primates elicited anti-CSP antibodies and also multifunctional CD4^+^ T cell responses [123]. Another approach used to target the* P. yoelii* CSP to DCs was to fuse its sequence to the macrophage inflammatory protein 3*α* (MIP3*α*) that targets the CCR6 receptor present on the surface of immature DCs. Mice were immunized with a DNA plasmid encoding the MIP3*α*-CSP in the presence of Vaxfectin, and protection was obtained against the challenge [124]. These results indicate that DCs may be targeted with* Plasmodium* antigens in an attempt to induce potent immune responses and ultimately induce long-lasting protection.

## 6. Concluding Remarks

The effects of* Plasmodium* infection on dendritic cells are broad and normally result in DC activation and induction of potent T cell responses that also lead to B cell activation and antibody production. However, evidence has been gathered that malaria may also suppress DC function. The complexity of the parasite cycle and also the different DCs subsets contribute to increase the level of difficulty in understanding the outcome of all interactions. A multidisciplinary approach to elucidate the mechanisms involved in the activation of DCs by* Plasmodium* is necessary. If we understand how the parasite modulates DCs, it may be possible to manipulate this information to develop an effective vaccine against malaria.

## References

[1] Nussenzweig M. C., Steinman R. M. (1980). Contribution of dendritic cells to stimulation of the murine syngeneic mixed leukocyte reaction. *Journal of Experimental Medicine*.

[2] Avery D. T., Kalled S. L., Ellyard J. I. (2003). BAFF selectively enhances the survival of plasmablasts generated from human memory B cells. *Journal of Clinical Investigation*.

[3] Ferlazzo G., Tsang M. L., Moretta L., Melioli G., Steinman R. M., Münz C. (2002). Human dendritic cells activate resting natural killer (NK) cells and are recognized via the NKp30 receptor by activated NK cells. *Journal of Experimental Medicine*.

[4] Gerosa F., Gobbi A., Zorzi P. (2005). The reciprocal interaction of NK cells with plasmacytoid or myeloid dendritic cells profoundly affects innate resistance functions. *The Journal of Immunology*.

[5] Reis e Sousa C. (2006). Dendritic cells in a mature age. *Nature Reviews Immunology*.

[6] Brocker T., Riedinger M., Karjalainen K. (1997). Targeted expression of major histocompatibility complex (MHC) class II molecules demonstrates that dendritic cells can induce negative but not positive selection of thymocytes in vivo. *Journal of Experimental Medicine*.

[7] Steinman R. M., Nussenzweig M. C. (2002). Avoiding horror autotoxicus: the importance of dendritic cells in peripheral T cell tolerance. *Proceedings of the National Academy of Sciences of the United States of America*.

[8] Iwasaki A., Medzhitov R. (2015). Control of adaptive immunity by the innate immune system. *Nature Immunology*.

[9] Kawai T., Akira S. (2010). The role of pattern-recognition receptors in innate immunity: update on toll-like receptors. *Nature Immunology*.

[10] Hoving J. C., Wilson G. J., Brown G. D. (2014). Signalling C-type lectin receptors, microbial recognition and immunity. *Cellular Microbiology*.

[11] Rathinam V. A. K., Vanaja S. K., Fitzgerald K. A. (2012). Regulation of inflammasome signaling. *Nature Immunology*.

[12] Wu J., Chen Z. J. (2014). Innate immune sensing and signaling of cytosolic nucleic acids. *Annual Review of Immunology*.

[13] Lemaitre B., Nicolas E., Michaut L., Reichhart J.-M., Hoffmann J. A. (1996). The dorsoventral regulatory gene cassette *spätzle/Toll/cactus* controls the potent antifungal response in Drosophila adults. *Cell*.

[14] Luger R., Valookaran S., Knapp N., Vizzardelli C., Dohnal A. M., Felzmann T. (2013). Toll-like receptor 4 engagement drives differentiation of human and murine dendritic cells from a pro- into an anti-inflammatory mode. *PLoS ONE*.

[15] Merad M., Sathe P., Helft J., Miller J., Mortha A. (2013). The dendritic cell lineage: ontogeny and function of dendritic cells and their subsets in the steady state and the inflamed setting. *Annual Review of Immunology*.

[16] Ginhoux F., Merad M. (2010). Ontogeny and homeostasis of Langerhans cells. *Immunology and Cell Biology*.

[17] Belz G. T., Shortman K., Bevan M. J., Heath W. R. (2005). CD8*α*+ dendritic cells selectively present MHC class I-restricted noncytolytic viral and intracellular bacterial antigens in vivo. *Journal of Immunology*.

[18] den Haan J. M. M., Lehar S. M., Bevan M. J. (2000). CD8^+^ but not CD8^−^ dendritic cells cross-prime cytotoxic T cells in vivo. *Journal of Experimental Medicine*.

[19] Vu Manh T.-P., Bertho N., Hosmalin A., Schwartz-Cornil I., Dalod M. (2015). Investigating evolutionary conservation of dendritic cell subset identity and functions. *Frontiers in Immunology*.

[20] Liu K., Nussenzweig M. C. (2010). Origin and development of dendritic cells. *Immunological Reviews*.

[21] Edelson B. T., Wumesh K. C., Juang R. (2010). Peripheral CD103^+^ dendritic cells form a unified subset developmentally related to CD8*α*
^+^ conventional dendritic cells. *The Journal of Experimental Medicine*.

[22] Nakano H., Yanagita M., Gunn M. D. (2001). CD11c^+^B220^+^Gr-1^+^ cells in mouse lymph nodes and spleen display characteristics of plasmacytoid dendritic cells. *Journal of Experimental Medicine*.

[23] Asselin-Paturel C., Boonstra A., Dalod M. (2001). Mouse type I IFN-producing cells are immature APCs with plasmacytoid morphology. *Nature Immunology*.

[24] O'Keeffe M., Hochrein H., Vremec D. (2002). Mouse plasmacytoid cells: long-lived cells, heterogeneous in surface phenotype and function, that differentiate into CD8- dendritic cells only after microbial stimulus. *Journal of Experimental Medicine*.

[25] Schlecht G., Garcia S., Escriou N., Freitas A. A., Leclerc C., Dadaglio G. (2004). Murine plasmacytoid dendritic cells induce effector/memory CD8^+^ T-cell responses in vivo after viral stimulation. *Blood*.

[26] Villadangos J. A., Young L. (2008). Antigen-presentation properties of plasmacytoid dendritic cells. *Immunity*.

[27] O'Doherty U., Peng M., Gezelter S. (1994). Human blood contains two subsets of dendritic cells, one immunologically mature and the other immature. *Immunology*.

[28] Dzionek A., Fuchs A., Schmidt P. (2000). BDCA-2, BDCA-3, and BDCA-4: three markers for distinct subsets of dendritic cells in human peripheral blood. *The Journal of Immunology*.

[29] Reizis B., Bunin A., Ghosh H. S., Lewis K. L., Sisirak V. (2011). Plasmacytoid dendritic cells: recent progress and open questions. *Annual Review of Immunology*.

[30] Nestle F. O., Zheng X.-G., Thompson C. B., Turka L. A., Nickoloff B. J. (1993). Characterization of dermal dendritic cells obtained from normal human skin reveals phenotypic and functionally distinctive subsets. *The Journal of Immunology*.

[31] Boltjes A., van Wijk F. (2014). Human dendritic cell functional specialization in steady-state and inflammation. *Frontiers in Immunology*.

[32] WHO (2014). World malaria report 2014. *WHO Report*.

[33] Amino R., Thiberge S., Martin B. (2006). Quantitative imaging of *Plasmodium* transmission from mosquito to mammal. *Nature Medicine*.

[34] Yamauchi Lucy L. M., Coppi A., Snounou G., Sinnis P. (2007). Plasmodium sporozoites trickle out of the injection site. *Cellular Microbiology*.

[35] Mota M. M., Pradel G., Vanderberg J. P. (2001). Migration of Plasmodium sporozoites through cells before infection. *Science*.

[36] Sturm A., Amino R., van de Sand C. (2006). Manipulation of host hepatocytes by the malaria parasite for delivery into liver sinusoids. *Science*.

[37] Bartoloni A., Zammarchi L. (2012). Clinical aspects of uncomplicated and severe malaria. *Mediterranean Journal of Hematology and Infectious Diseases*.

[38] Taylor W. R. J., Hanson J., Turner G. D. H., White N. J., Dondorp A. M. (2012). Respiratory manifestations of malaria. *Chest*.

[39] Miller L. H., Baruch D. I., Marsh K., Doumbo O. K. (2002). The pathogenic basis of malaria. *Nature*.

[40] Barnwell J. W., Asch A. S., Nachman R. L., Yamaya M., Aikawa M., Ingravallo P. (1989). A human 88-kD membrane glycoprotein (CD36) functions in vitro as a receptor for a cytoadherence ligand on *Plasmodium falciparum*-infected erythrocytes. *The Journal of Clinical Investigation*.

[41] Berendt A. R., Simmons D. L., Tansey J., Newbold C. I., Marsh K. (1989). Intercellular adhesion molecule-1 is an endothelial cell adhesion receptor for *Plasmodium falciparum*. *Nature*.

[42] Rowe J. A., Claessens A., Corrigan R. A., Arman M. (2009). Adhesion of *Plasmodium falciparum*-infected erythrocytes to human cells: molecular mechanisms and therapeutic implications. *Expert Reviews in Molecular Medicine*.

[43] Bull P. C., Lowe B. S., Kortok M., Molyneux C. S., Newbold C. I., Marsh K. (1998). Parasite antigens on the infected red cell surface are targets for naturally acquired immunity to malaria. *Nature Medicine*.

[44] Stevenson M. M., Riley E. M. (2004). Innate immunity to malaria. *Nature Reviews Immunology*.

[45] Baird J. K., Jones T. R., Danudirgo E. W. (1991). Age-dependent acquired protection against *Plasmodium falciparum* in people having two years exposure to hyperendemic malaria. *The American Journal of Tropical Medicine and Hygiene*.

[46] Egan A. F., Morris J., Barnish G. (1996). Clinical immunity to *Plasmodium falciparum* malaria is associated with serum antibodies to the 19-kDa C-terminal fragment of the merozoite surface antigen, PfMSP-1. *Journal of Infectious Diseases*.

[47] Webster D., Hill A. V. S. (2003). Progress with new malaria vaccines. *Bulletin of the World Health Organization*.

[48] Wykes M. N., Good M. F. (2008). What really happens to dendritic cells during malaria?. *Nature Reviews Microbiology*.

[49] Inoue S.-I., Niikura M., Mineo S., Kobayashi F. (2013). Roles of IFN-*γ* and *γδ* T cells in protective immunity against blood-stage malaria. *Frontiers in Immunology*.

[50] Weiss W. R., Jiang C. G. (2012). Protective CD8+ T lymphocytes in primates immunized with malaria sporozoites. *PLoS ONE*.

[51] Weiss W. R., Sedegah M., Beaudoin R. L., Miller L. H., Good M. F. (1988). CD8^+^ T cells (cytotoxic/suppressors) are required for protection in mice immunized with malaria sporozoites. *Proceedings of the National Academy of Sciences of the United States of America*.

[52] Tsuji M. (2010). A retrospective evaluation of the role of T cells in the development of malaria vaccine. *Experimental Parasitology*.

[53] Wizel B., Houghten R. A., Parker K. C. (1995). Irradiated sporozoite vaccine induces HLA-B8-restricted cytotoxic T lymphocyte responses against two overlapping epitopes of the Plasmodium falciparum sporozoite surface protein 2. *Journal of Experimental Medicine*.

[54] Butler N. S., Schmidt N. W., Harty J. T. (2010). Differential effector pathways regulate memory CD8 T cell immunity against *Plasmodium berghei* versus *P. yoelii* sporozoites. *The Journal of Immunology*.

[55] Perez-Mazliah D., Langhorne J. (2014). CD4 T-cell subsets in malaria: Th1/Th2 revisited. *Frontiers in Immunology*.

[56] Banchereau J., Steinman R. M. (1998). Dendritic cells and the control of immunity. *Nature*.

[57] Urban B. C., Ferguson D. J. P., Pain A. (1999). Plasmodium falciparuminfected erythrocytes modulate the maturation of dendritic cells. *Nature*.

[58] Elliott S. R., Spurck T. P., Dodin J. M. (2007). Inhibition of dendritic cell maturation by malaria is dose dependent and does not require *Plasmodium falciparum* erythrocyte membrane protein 1. *Infection and Immunity*.

[59] Urban B. C., Cordery D., Shafi M. J. (2006). The frequency of BDCA3-positive dendritic cells is increased in the peripheral circulation of Kenyan children with severe malaria. *Infection and Immunity*.

[60] Guermonprez P., Helft J., Claser C. (2013). Inflammatory Flt3l is essential to mobilize dendritic cells and for T cell responses during Plasmodium infection. *Nature Medicine*.

[61] Urban B. C., Shafi M. J., Cordery D. V. (2006). Frequencies of peripheral blood myeloid cells in healthy Kenyan children with alpha+ thalassemia and the sickle cell trait. *The American Journal of Tropical Medicine and Hygiene*.

[62] Gonçalves R. M., Salmazi K. C., Santos B. A. N. (2010). CD4+ CD25+ Foxp3+ regulatory T cells, dendritic cells, and circulating cytokines in uncomplicated malaria: do different parasite species elicit similar host responses?. *Infection and Immunity*.

[63] Jangpatarapongsa K., Chootong P., Sattabongkot J. (2008). *Plasmodium vivax* parasites alter the balance of myeloid and plasmacytoid dendritic cells and the induction of regulatory T cells. *European Journal of Immunology*.

[64] Pinzon-Charry A., Woodberry T., Kienzle V. (2013). Apoptosis and dysfunction of blood dendritic cells in patients with falciparum and vivax malaria. *The Journal of Experimental Medicine*.

[65] Kho S., Marfurt J., Noviyanti R. (2015). Preserved dendritic cell HLA-DR expression and reduced regulatory T cell activation in Asymptomatic Plasmodium falciparum and P. vivax infection. *Infection and Immunity*.

[66] Vichchathorn P., Jenwithisuk R., Leelaudomlipi S. (2006). Induction of specific immune responses against the *Plasmodium vivax* liver-stage via in vitro activation by dendritic cells. *Parasitology International*.

[67] Bruña-Romero O., Rodriguez A. (2001). Dendritic cells can initiate protective immune responses against malaria. *Infection and Immunity*.

[68] Jung S., Unutmaz D., Wong P. (2002). In vivo depletion of CD11c^+^ dendritic cells abrogates priming of CD8^+^ T cells by exogenous cell-associated antigens. *Immunity*.

[69] Chakravarty S., Cockburn I. A., Kuk S., Overstreet M. G., Sacci J. B., Zavala F. (2007). CD8+ T lymphocytes protective against malaria liver stages are primed in skin-draining lymph nodes. *Nature Medicine*.

[70] Radtke A. J., Kastenmüller W., Espinosa D. A. (2015). Lymph-node resident CD8*α*
^+^ dendritic cells capture antigens from migratory malaria sporozoites and induce CD8^+^ T cell responses. *PLoS Pathogens*.

[71] Plebanski M., Hannan C. M., Behboudi S. (2005). Direct processing and presentation of antigen from malaria sporozoites by professional antigen-presenting cells in the induction of CD8+ T-cell responses. *Immunology and Cell Biology*.

[72] Behboudi S., Moore A., Hill A. V. S. (2004). Splenic dendritic cell subsets prime and boost CD8 T cells and are involved in the generation of effector CD8 T cells. *Cellular Immunology*.

[73] Murray S. A., Mohar I., Miller J. L. (2015). CD40 is required for protective immunity against liver stage *Plasmodium* infection. *Journal of Immunology*.

[74] Ocaña-Morgner C., Mota M. M., Rodriguez A. (2003). Malaria blood stage suppression of liver stage immunity by dendritic cells. *The Journal of Experimental Medicine*.

[75] Orengo J. M., Wong K. A., Ocãa-Morgner C., Rodriguez A. (2008). A Plasmodium yoelii soluble factor inhibits the phenotypic maturation of dendritic cells. *Malaria Journal*.

[76] Wilson N. S., Behrens G. M. N., Lundie R. J. (2006). Systemic activation of dendritic cells by Toll-like receptor ligands or malaria infection impairs cross-presentation and antiviral immunity. *Nature Immunology*.

[77] Lundie R. J., de Koning-Ward T. F., Davey G. M. (2008). Blood-stage Plasmodium infection induces CD8^+^ T lymphocytes to parasite-expressed antigens, largely regulated by CD8*α*
^+^ dendritic cells. *Proceedings of the National Academy of Sciences of the United States of America*.

[78] Perry J. A., Rush A., Wilson R. J., Olver C. S., Avery A. C. (2004). Dendritic cells from malaria-infected mice are fully functional APC. *The Journal of Immunology*.

[79] Luyendyk J., Olivas O. R., Ginger L. A., Avery A. C. (2002). Antigen-presenting cell function during *Plasmodium yoelii* infection. *Infection and Immunity*.

[80] Seixas E., Cross C., Quin S., Langhorne J. (2001). Direct activation of dendritic cells by the malaria parasite, *Plasmodium chabaudi chabaudi*. *European Journal of Immunology*.

[81] Leisewitz A. L., Rockett K. A., Gumede B., Jones M., Urban B., Kwiatkowski D. P. (2004). Response of the splenic dendritic cell population to malaria infection. *Infection and Immunity*.

[82] Sponaas A.-M., Cadman E. T., Voisine C. (2006). Malaria infection changes the ability of splenic dendritic cell populations to stimulate antigen-specific T cells. *Journal of Experimental Medicine*.

[83] Sponaas A.-M., Belyaev N., Falck-Hansen M., Potocnik A., Langhorne J. (2012). Transient deficiency of dendritic cells results in lack of a merozoite surface protein 1-specific CD4 T cell response during peak *Plasmodium chabaudi* blood-stage infection. *Infection and Immunity*.

[84] Langhorne J., Ndungu F. M., Sponaas A.-M., Marsh K. (2008). Immunity to malaria: more questions than answers. *Nature Immunology*.

[85] Voisine C., Mastelic B., Sponaas A.-M., Langhorne J. (2010). Classical CD11c+ dendritic cells, not plasmacytoid dendritic cells, induce T cell responses to Plasmodium chabaudi malaria. *International Journal for Parasitology*.

[86] Wong K. A., Rodriguez A. (2008). *Plasmodium* infection and endotoxic shock induce the expansion of regulatory dendritic cells. *Journal of Immunology*.

[87] Ing R., Segura M., Thawani N., Tam M., Stevenson M. M. (2006). Interaction of mouse dendritic cells and malaria-infected erythrocytes: Uptake, maturation, and antigen presentation. *Journal of Immunology*.

[88] Borges da Silva H., Fonseca R., Cassado A. D. A. (2015). In vivo approaches reveal a key role for DCs in CD4+ T cell activation and parasite clearance during the acute phase of experimental blood-stage malaria. *PLoS Pathogens*.

[89] de Walick S., Amante F. H., McSweeney K. A. (2007). Cutting edge: conventional dendritic cells are the critical APC required for the induction of experimental cerebral malaria. *The Journal of Immunology*.

[90] Tamura T., Kimura K., Yui K., Yoshida S. (2015). Reduction of conventional dendritic cells during Plasmodium infection is dependent on activation induced cell death by type I and II interferons. *Experimental Parasitology*.

[91] Liu X. Q., Stacey K. J., Horne-Debets J. M. (2012). Malaria infection alters the expression of B-cell activating factor resulting in diminished memory antibody responses and survival. *European Journal of Immunology*.

[92] Ryg-Cornejo V., Nie C. Q., Bernard N. J. (2013). NK cells and conventional dendritic cells engage in reciprocal activation for the induction of inflammatory responses during *Plasmodium berghei* ANKA infection. *Immunobiology*.

[93] Loharungsikul S., Troye-Blomberg M., Amoudruz P. (2008). Expression of Toll-like receptors on antigen-presenting cells in patients with falciparum malaria. *Acta Tropica*.

[94] Pichyangkul S., Yongvanitchit K., Kum-Arb U. (2004). Malaria blood stage parasites activate human plasmacytoid dendritic cells and murine dendritic cells through a toll-like receptor 9-dependent pathway. *Journal of Immunology*.

[95] Franklin B. S., Rodrigues S. O., Antonelli L. R. (2007). MyD88-dependent activation of dendritic cells and CD4^+^ T lymphocytes mediates symptoms, but is not required for the immunological control of parasites during rodent malaria. *Microbes and Infection*.

[96] Gazzinelli R. T., Kalantari P., Fitzgerald K. A., Golenbock D. T. (2014). Innate sensing of malaria parasites. *Nature Reviews Immunology*.

[97] Jani D., Nagarkatti R., Beatty W. (2008). HDP—a novel heme detoxification protein from the malaria parasite. *PLoS Pathogens*.

[98] Schwarzer E., Alessio M., Ulliers D., Arese P. (1998). Phagocytosis of the malarial pigment, hemozoin, impairs expression of major histocompatibility complex class II antigen, CD54, and CD11c in human monocytes. *Infection and Immunity*.

[99] Skorokhod O. A., Alessio M., Mordmüller B., Arese P., Schwarzer E. (2004). Hemozoin (malarial pigment) inhibits differentiation and maturation of human monocyte-derived dendritic cells: a peroxisome proliferator-activated receptor-*γ*-mediated effect. *Journal of Immunology*.

[100] Skorokhod O., Schwarzer E., Grune T., Arese P. (2005). Role of 4-hydroxynonenal in the hemozoin-mediated inhibition of differentiation of human monocytes to dendritic cells induced by GM-CSF/IL-4. *BioFactors*.

[101] Giusti P., Urban B. C., Frascaroli G. (2011). *Plasmodium falciparum*-infected erythrocytes and *β*-hematin induce partial maturation of human dendritic cells and increase their migratory ability in response to lymphoid chemokines. *Infection and Immunity*.

[102] Bujila I., Schwarzer E., Skorokhod O., Weidner J. M., Troye-Blomberg M., Östlund Farrants A. (2016). Malaria-derived hemozoin exerts early modulatory effects on the phenotype and maturation of human dendritic cells. *Cellular Microbiology*.

[103] Millington O. R., Di Lorenzo C., Phillips R. S., Garside P., Brewer J. M. (2006). Suppression of adaptive immunity to heterologous antigens during *Plasmodium* infection through hemozoin-induced failure of dendritic cell function. *Journal of Biology*.

[104] Millington O. R., Gibson V. B., Rush C. M. (2007). Malaria impairs T cell clustering and immune priming despite normal signal 1 from dendritic cells. *PLoS Pathogens*.

[105] Coban C., Ishii K. J., Sullivan D. J., Kumar N. (2002). Purified malaria pigment (hemozoin) enhances dendritic cell maturation and modulates the isotype of antibodies induced by a DNA vaccine. *Infection and Immunity*.

[106] Coban C., Ishii K. J., Kawai T. (2005). Toll-like receptor 9 mediates innate immune activation by the malaria pigment hemozoin. *The Journal of Experimental Medicine*.

[107] Parroche P., Lauw F. N., Goutagny N. (2007). Malaria hemozoin is immunologically inert but radically enhances innate responses by presenting malaria DNA to Toll-like receptor 9. *Proceedings of the National Academy of Sciences of the United States of America*.

[108] Wu X., Gowda N. M., Kumar S., Gowda D. C. (2010). Protein-DNA complex is the exclusive malaria parasite component that activates dendritic cells and triggers innate immune responses. *Journal of Immunology*.

[109] Shio M. T., Eisenbarth S. C., Savaria M. (2009). Malarial hemozoin activates the NLRP3 inflammasome through Lyn and Syk kinases. *PLoS Pathogens*.

[110] Dostert C., Guarda G., Romero J. F. (2009). Malarial hemozoin is a Nalp3 inflammasome activating danger signal. *PLoS ONE*.

[111] Sharma S., DeOliveira R. B., Kalantari P. (2011). Innate immune recognition of an AT-rich stem-loop DNA motif in the Plasmodium falciparum genome. *Immunity*.

[112] Tachado S. D., Gerold P., McConville M. J. (1996). Glycosylphosphatidylinositol toxin of *Plasmodium* induces nitric oxide synthase expression in macrophages and vascular endothelial cells by a protein tyrosine kinase-dependent and protein kinase C-dependent signaling pathway. *Journal of Immunology*.

[113] Zhu J., Krishnegowda G., Gowda D. C. (2005). Induction of proinflammatory responses in macrophages by the glycosylphosphatidylinositols of *Plasmodium falciparum*: the requirement of extracellular signal-regulated kinase, p38, c-Jun N-terminal kinase and NF-*κ*B pathways for the expression of proinflammatory cytokines and nitric oxide. *The Journal of Biological Chemistry*.

[114] Krishnegowda G., Hajjar A. M., Zhu J. (2005). Induction of proinflammatory responses in macrophages by the glycosylphosphatidylinositols of *Plasmodium falciparum*: cell signaling receptors, glycosylphosphatidylinositol (GPI) structural requirement, and regulation of GPI activity. *The Journal of Biological Chemistry*.

[115] Erdman L. K., Cosio G., Helmers A. J., Gowda D. C., Grinstein S., Kain K. C. (2009). CD36 and TLR interactions in inflammation and phagocytosis: implications for malaria. *The Journal of Immunology*.

[116] Kumar S., Gowda N. M., Wu X., Gowda R. N., Gowda D. C. (2012). CD36 modulates proinflammatory cytokine responses to Plasmodium falciparum glycosylphosphatidylinositols and merozoites by dendritic cells. *Parasite Immunology*.

[117] Togbe D., Schofield L., Grau G. E. (2007). Murine cerebral malaria development is independent of toll-like receptor signaling. *The American Journal of Pathology*.

[118] Lepenies B., Cramer J. P., Burchard G. D., Wagner H., Kirschning C. J., Jacobs T. (2008). Induction of experimental cerebral malaria is independent of TLR2/4/9. *Medical Microbiology and Immunology*.

[119] Coban C., Uematsu S., Arisue N. (2007). Pathological role of Toll-like receptor signaling in cerebral malaria. *International Immunology*.

[120] Maglinao M., Klopfleisch R., Seeberger P. H., Lepenies B. (2013). The C-type lectin receptor DCIR is crucial for the development of experimental cerebral malaria. *Journal of Immunology*.

[121] van de Hoef D. L., Coppens I., Holowka T., Ben Mamoun C., Branch O., Rodriguez A. (2013). *Plasmodium falciparum*-derived uric acid precipitates induce maturation of dendritic cells. *PLoS ONE*.

[122] Boscardin S. B., Hafalla J. C. R., Masilamani R. F. (2006). Antigen targeting to dendritic cells elicits long-lived T cell help for antibody responses. *Journal of Experimental Medicine*.

[123] Tewari K., Flynn B. J., Boscardin S. B. (2010). Poly(I:C) is an effective adjuvant for antibody and multi-functional CD4+ T cell responses to *Plasmodium falciparum* circumsporozoite protein (CSP) and *α*DEC-CSP in non human primates. *Vaccine*.

[124] Luo K., Zhang H., Zavala F., Biragyn A., Espinosa D. A., Markham R. B. (2014). Fusion of antigen to a dendritic cell targeting chemokine combined with adjuvant yields a malaria dna vaccine with enhanced protective capabilities. *PLoS ONE*.

[125] Ginhoux F., Liu K., Helft J. (2009). The origin and development of nonlymphoid tissue CD103^+^ DCs. *The Journal of Experimental Medicine*.

[126] Segura E., Valladeau-Guilemond J., Donnadieu M.-H., Sastre-Garau X., Soumelis V., Amigorena S. (2012). Characterization of resident and migratory dendritic cells in human lymph nodes. *Journal of Experimental Medicine*.

